# Women in psychiatry: A view from the Indian subcontinent

**DOI:** 10.4103/0019-5545.55088

**Published:** 2009

**Authors:** Mamta Sood, Rakesh Kumar Chadda

**Affiliations:** Department of Psychiatry, All India Institute of Medical Sciences, Ansari Nagar, New Delhi - 110 029, India

**Keywords:** Women, psychiatry, Indian subcontinent

## Abstract

**Background:**

Psychiatry has not been a preferred medical specialty for women in the Indian subcontinent unlike in the Western countries like USA, Canada or UK. Recent years have seen an increase in the number of women doctors in India choosing psychiatry as career.

**Materials and Methods:**

Information on women in psychiatry in the Indian subcontinent was collected using resources like PubMed, directories of the professional societies, websites of medical institues, souvenirs and scientific programme of various conferences and personal communication with psychiatrists, and the data about postgraduate trainees available with the authors' own institute.

**Results:**

Women psychiatrists constitute about 15% of total psychiatrists in India, out of whom only 10% are at a relatively senior level, and the most are young. The women psychiatrists are also in faculty positions in a number of medical schools and have held important positions in the Indian Psychiatric Society at different times.

**Conclusions:**

Most of the women psychiatrists appear to be still at junior levels, having joined the profession relatively recently as compared to their male counterparts. The trend at increasing number of women psychiatrists in the Indian subcontinent is similar to the worldwide trends.

## INTRODUCTION

In the Indian subcontinent, the medical profession is considered noble, prestigious, and well paid. The profession has often been held in high esteem as a career for women, considering the physician's role as a healer as well as a nurturer. However, the socio-cultural norms expect women to be primarily responsible for domestic, family and child related duties. These duties need to be integrated with the professional duties. Because of the expectation of multiple roles, majority of the women in the medical profession often choose basic sciences or the discipline of obstetrics and gynecology for specialization. The former are preferred due to the fixed timing schedule and absence of emergency duties, and the latter because women patients in the subcontinent often prefer to be examined by women physicians. Psychiatry in general is still not been considered an important medical specialty due to various societal apprehensions and ignorance. Even the larger medical fraternity perceives its origins more in magic, Karma or distorted spirituality. The specialty continues to be associated with unpredictable and violent patients and therefore is not considered specialty of choice for most of the women. However, a career in psychiatry allows the person to be able to do justice to multiple roles in a better way than the other specialties because of possibility of regular hours and relative flexibility of working schedule.[1]

Even in the west, till 1970s, a lone woman would be seen in psychiatry residency courses.[2] However, the trends have changed since then and women physicians joining psychiatry has been on the rise in the high income countries. For example, in the USA, psychiatry has the fourth highest number of women specialists and 40-45% of first-year residents in psychiatry are women.[3] In the UK and Ireland, women form 45% of basic specialist trainees and 48% of higher specialist trainees in psychiatry.[4] In Canada, the percentage of women psychiatric residents increased from 23.5% to 43.4% over a period of 10 years from 1970s to 1980s.[5]

Though there is plenty of data available from the high-income countries on this issue, no such data is available from low-income countries like India. The paper discusses about the issues related to women psychiatrists from the Indian subcontinent, as a representative of low-income countries.

## MATERIALS AND METHODS

Literature search was done on PUBMED using key words: women physicians, women psychiatrists, female physicians, female psychiatrists, faculty, pregnancy, maternity leave, research, publications, Bangladesh, Bhutan, India, Maldives, Nepal, Pakistan and Sri Lanka, and manual search of Indian and international journals available in the local libraries. However, no studies could be retrieved. As no systematic data was available on this issue, a preliminary manual search was done from sources like membership directory of the Indian Psychiatric Society (2006), database of the Medical Council of India, souvenirs and scientific programme of various conferences and personal communication with psychiatrists, and the data about postgraduate trainees available with the authors' own institute.

## RESULTS

In India, 19,214 medical graduates are trained annually. Postgraduate training in psychiatry is of three years duration for degree course and two years for diploma course after graduation. Every year, 134 posts are available for postgraduate degree and 96 for postgraduate diploma in psychiatry.[6] But no information is available on how many women have joined the posts in the previous years. Women have formed 22% and 20% of the intake in undergraduate and postgraduate medical courses, respectively in the last 5 years at the All India Institute of Medical Sciences, New Delhi (authors' institution), one of the premier medical institutions of the country. Women formed about 20% of the candidates joining psychiatry in the last 5 years. However, it is not known how many women candidates join psychiatry residency in the country.

Indian Psychiatric Society, the national body of psychiatrists in India had a membership of 2829 in 2006, out of which 14.6% are women and 85.4% are men. A member of the society becomes eligible for fellow after 5 years post qualification experience in the psychiatry. The society has 1830 fellows and 999 ordinary members. Women constitute about 10% of the fellows and 20% of the ordinary members. Currently, there is no female office bearer in the society and only one of the council members is a woman. Since its inception in 1947, women psychiatrists were presidents only thrice, in 1981, 1983 and 1990, and held post of secretary only twice in 1967 and 1968. Though the society has been publishing its journal, the Indian Journal of Psychiatry for more than 50 years, it never had a woman editor. In the current editorial board, there is no woman psychiatrist and only one is in the journal committee. No woman psychiatrist holds a post as advisor on policy matters related to mental health in general or in relation to women to the Government of India.

As per information available from the Medical Council of India, there are 837 faculty members in psychiatry in various medical schools of India.[6] But the number of female faculty members in psychiatry is not known. An attempt was made to collect data from websites of some of premier institutes. At the All India Institute of Medical Sciences, New Delhi, the ratio of female to male faculty is 4:9; none of the women is a professor. At the Post Graduate Institute of Medical Education and Research, Chandigarh, the ratio is 1:7, one is professor. At the National Institute of Mental Health and Neurosciences, Bangalore, ratio is 7:20, 4 are professors. It is not known, how many have been heads of departments of psychiatry in various parts of the country, only handful could be counted. A few women have been known to have headed a psychiatric hospital or a medical school.

There are no guidelines for pregnancy and maternity leave for women psychiatric residents from the Medical Council of India. A resident has to attend not less than 80% of the training during each calendar year.[6] If pregnancy occurs, either the resident has to manage within the permitted time of not attending or has to postpone her examination. If she joins a government sector job, there is a provision for maternity leave of 135 days.[7]

But no hospital or medical college provides reliable onsite daycare for children. If job is discontinued due to any reason including issues like pregnancy or childcare, options for revival of career after a certain period are presently unavailable due to restrictions in age and qualification and no system or policies from government at present address these issues.[8]

## DISCUSSION

Women psychiatrists constitute about 15% of total psychiatrists in India, out of whom only 10% are at a relatively senior level, and the most are young. It is possible that the number of women joining psychiatry is increasing in the recent years, which is responsible for a higher percentage of them at junior oppositions. The trend at increasing number of women psychiatrists in India is similar to the worldwide trends. The change may be recent in India, though it has existed worldwide for the last few decades. Situation in the other countries in the subcontinent is likely to be similar, though no data is available.

In the last 30-40 years, a number of new departments of psychiatry have come up in the various general hospitals in India. There has also been downsizing of some mental hospitals with more emphasis on general hospital and community psychiatry. Office based practice of psychiatry has also come up especially in the big cities in the country. All these factors have led to a general acceptance of the specialty in the mainstream, and also attraction of women doctors to the discipline. Women psychiatrists are represented in different sectors like general hospital psychiatric units, psychiatric hospitals and the office based practice. However, most of the women psychiatrists appear to be still at junior levels, having joined the profession relatively recently as compared to their male counterparts. Exact figures of the gender based distribution of psychiatrists at different job levels are not available. Data available from one premier medical institution of the country has shown the percentage of women joining psychiatry in the last 5 years is not different from the total percentage of women doctors joining the institute for post graduation.

The women psychiatrists are in faculty positions in a number of medical schools and also holding the departmental chairs at a few places, but their exact number is not known. Similarly, a few have held important positions in the Indian Psychiatric Society, the national body of psychiatrists in India, but their overall number is negligible as compared to the men. Partly due to their own inhibitions and partly subtle gender biases, they shun taking positions as office bearers on committees. The role of women psychiatrists in policy making of the specialty therefore remains negligible.

However, even now, most of the medical institutions remain oriented toward traditional families of the past, rather than today's dual-career parents, with rare availability of onsite daycare. There is little opportunity for flexible training, creative scheduling of job or training if they want to come back to academics after a gap. There is still some prejudice towards offering the top positions to the women, though many of the countries in the Indian subcontinent had women prime ministers and the current President of India is also a woman.

In the West, women psychiatrists as a group have been reported to lag behind their men colleagues in attaining positions of authority and leadership in academics, professional organizations, and medical institutions. In USA, women only constitute 29% of associate professors, 15% of full professors, and 6% of department chairs in medical schools.[9] In England also, women psychiatrists significantly are less likely to pursue an academic career and professional position than men.[10] Women psychiatrists have been reported to have poorer coping skills, more physical and emotional symptoms, and are more likely to report stress, anxiety, and depression.[11] The situation has not been studied formally in India or other developing countries, reason could be that their number was often too small.

Working style of the women doctors has been found to differ from their male colleagues in some aspects. Many women psychiatrists are astute in their understanding of psychology, and are more empathic in approach. Their patients report better satisfaction levels as they are more likely to engage patients as active partners in their care by adopting a democratic style of communication. They spend a significantly greater proportion of time on preventive services and counseling, compared to their male colleagues.[12] Most women psychiatrists appear extremely interested and glad of their choice to be in this field. Young women medicos taking psychiatry as a career should be encouraged to join the specialty. The specialty also vibes with their own biological ‘persona’ and this helps in greater healing of those suffering from mental anguish. Their increasing number will also bring a varied perspective on mental health issues, diversity of work styles and values. Inclusion of women psychiatrists in education, training, research, clinical care, and policymaking will have influence on the mental health care system. The profession needs to innovate ways of maximizing optimal use of this substantial talent pool and intellectual capital.

## CONCLUSIONS

There appears to be an increase in the number of female psychiatrists in India in the recent past and apparently also in other countries in the Indian subcontinent. Many of the concerns faced by women psychiatrists are reflection of the concerns faced by women physicians. They need support systems both at home and in workplace to succeed in concurrent personal, family, and professional roles. In the high-income countries, despite their increasing numbers, they have not advanced in their careers like their men colleagues. They are also likely to face similar problems as in the West and as faced by their colleagues in the other medical disciplines in the country. The specific issues and concerns related to women psychiatrists in the low-income countries need to be studied systematically.
